# Temperature Tolerance of Self-Assembled Gels and Viscoelastic Solutions of Wormlike Micelles of Potassium Oleate Induced by Embedded Cellulose Nanocrystals

**DOI:** 10.3390/gels12060459

**Published:** 2026-05-24

**Authors:** Mikhail M. Avdeev, Vyacheslav S. Molchanov, Alexander L. Kwiatkowski, Yuri M. Chesnokov, Akhmed Kh. Islamov, Kuanysh Nazarov, Olga E. Philippova

**Affiliations:** 1 Physics Department, Lomonosov Moscow State University, Moscow 119991, Russia; avdeev@polly.phys.msu.ru (M.M.A.); molchan@polly.phys.msu.ru (V.S.M.); kvyatkovskij@physics.msu.ru (A.L.K.); 2 Frank Laboratory of Neutron Physics, Joint Institute for Nuclear Research, Dubna 141980, Russia; islamov@nf.jinr.ru (A.K.I.);; 3 National Research Center «Kurchatov Institute», Moscow 123182, Russia; chessyura@yandex.ru; 4 Institute of Nuclear Physics, Almaty 050032, Kazakhstan

**Keywords:** cellulose nanocrystals, wormlike surfactant micelles, rheological properties, potassium oleate

## Abstract

Wormlike micelles (WLMs) of surfactants are widely used as smart thickeners in various applications, including enhanced oil recovery. However, their thickening ability needs to be improved both at ambient and elevated temperatures. In the present paper, we propose to enhance the viscoelastic properties of surfactant solutions by incorporating carboxymethylated cellulose nanocrystals (CNCs). Upon addition of CNCs, dilute solutions of short WLMs acquire viscoelasticity and then transition into a viscoelastic solid state. This process is accompanied by an increase in the viscosity and storage modulus by up to five and four orders of magnitude, respectively. The observed effect of CNCs on the storage modulus and viscosity is greater than that of any of the previously studied WLM-CNC systems. It is attributed to the formation of a network of fibrillar-like aggregates composed of WLMs and CNCs, which was confirmed by cryo-TEM data. To our knowledge, such kind of aggregates have not been observed before. When CNCs are added to a transient network of long entangled WLMs, the viscoelastic solution transitions into a viscoelastic solid state, which results in an increase in the viscosity and storage modulus by up to two orders of magnitude. CNCs provide the WLM solution with greater resistance to heating, such that the storage modulus remains almost unchanged when the temperature increases from 20 to 70 °C. Moreover, a heat-induced gelation was observed. It was shown that higher concentrations of nanocrystals lower the critical gel temperature, indicating that they promote the gelation of the mixture. SANS data revealed that the local structures of both micelles and nanocrystals are preserved in the mixed system upon heating. According to ITC data, at room temperature, the interaction between surfactant ions and similarly charged nanocrystals is governed by both enthalpy and entropy, which suggests that hydrogen bonding plays a major role in this process, although hydrophobic interactions may also be involved. When the temperature increases to 60 °C, the aggregation becomes entropy-driven, indicating that hydrophobic interactions begin to dominate. The results obtained can expand the range of practical applications of WLMs as thickening agents, in particular, to higher-temperature conditions in deeper oil wells.

## 1. Introduction

Wormlike micelles (WLMs) formed by surfactant molecules are of current interest due to their unique properties. Being a few micrometers in length but thin at the same time, WLMs are able to form an entangled network with high viscoelastic properties [1,2,3,4,5]. These properties are widely exploited in various applications of WLMs as thickening agents, particularly in fracturing fluids in the oil industry [6], but also in personal care and consumer products [7].

However, WLM solutions and hydrogels suffer from a limited operating temperature range. This is due to their self-assembled nature, which makes the viscoelastic properties sensitive to temperature, which affects the structure of the surfactant aggregates [8,9]. Usually, heating causes the shortening of micelles [1], which finally results in a loss of viscoelasticity when the micelles become so short that their transient network breaks down [2,10].

One of the potential solutions to this issue could be the strengthening of the WLM network structure by nanoparticles [11,12,13,14,15,16,17,18,19,20,21,22,23,24,25,26,27,28,29,30,31]. Inorganic nanoparticles are known to crosslink WLMs [14,15] or promote their elongation [16]. The interaction of nanoparticles with WLMs proceeds via a fusion of energetically unfavorable end caps of micelles and the surfactant layer on the surface of nanoparticles [14,17]. The end caps of micelles are suggested to be primarily involved in this interaction, as the semi-spherical arrangement of surfactant in these regions is less favorable compared to the cylindrical one in the main body of the micellar chains [16]. The linking between WLMs and particles was observed using freeze-fracture [18] and cryo-electron microscopy [14,19] techniques and was confirmed by coarse-grained molecular dynamics simulations [20,21].

The enhancement in viscoelasticity in WLM solutions and gels by inorganic nanoparticles was demonstrated using nanoparticles of various shapes and compositions, including silica nanospheres [11,22], halloysite nanotubes [23], multiwalled carbon nanotubes [12], clay nanosheets [19] and others. The increase in viscosity caused by nanoparticles is often more significant at elevated temperatures, as the shorter micelles have more end caps available for interaction with the nanoparticles [31]. This makes nanoparticles very promising for increasing the temperature tolerance of WLMs.

Recently, a new trend has emerged that focuses on the use of organic nanoparticles made from biopolymers [24,25,26,27,28,29,30] as reinforcing fillers of WLM gels and solutions instead of inorganic ones. Among organic nanoparticles, nano-scaled cellulose is a particularly promising option. Cellulose-based nanoparticles are produced from natural and renewable raw materials such as wood, crops, cellulosic agriculture/food waste, and so on [32]. The advantage of such objects consists of their biodegradability, non-toxicity and low cost. One of the most promising types of cellulose-based materials for strengthening the WLM network is cellulose nanocrystals (CNCs). They are rigid rods that are several hundred nanometers in length [33,34,35], with a high degree of crystallinity (up to 90%) [36,37]. CNCs have superlative mechanical properties (the elastic modulus of 110–220 GPa, the tensile strength of 7.5–7.7 GPa [38]), which make them the filler material of choice for reinforcement of WLM solutions and gels.

To date, only two papers have been published on the influence of CNCs on the rheology of WLMs at different temperatures [28,29]. In one of these papers [29], it was found that CNCs cause an increase in the viscosity of WLMs formed by zwitterionic surfactant erucyl dimethyl amidopropyl betaine and nonionic surfactant alkyl polyglucoside. This effect was attributed to the formation of the entanglements between WLMs and CNCs. However, even after adding CNCs, viscosity still decreases when heating from 25 to 90 °C. In the second paper [28], it was shown that CNCs can not only enhance the viscosity and elastic modulus of WLM solutions by more than one order of magnitude, but they also induce the formation of hydrogels. An important finding of this study was that the viscoelastic properties of WLMs reinforced with CNCs remained unchanged upon heating from 20 to 55 °C. This behavior was explained by the electrostatic interactions and hydrogen bonding between WLMs and CNCs, as shown by Fourier-transform infrared spectroscopy (FTIR) data, similar to what has been observed for WLMs and cellulose nanofibers in previous studies [27,30]. It can be suggested that hydrophobic interactions may also contribute to this effect. However, the experiments conducted so far have not allowed one to determine their contribution. This could be done, for instance, by isothermal titration calorimetry (ITC) [39]. Also, there is still a lack of structural studies on WLM-CNC systems, which would reveal their morphology. The experiments in the second paper [28] were conducted using a sophisticated anionic surfactant containing a bulky hydrophobic part with a tricyclic hydrophenanthrene skeleton and an n-hexadecyl tail. This surfactant was obtained from pine tree rosin through a three-stage synthesis [28]. To prepare WLMs, it was mixed with cationic surfactant cetyltrimethylammonium bromide (CTAB). The use of a poorly available surfactant limits the practical applications of the temperature resistance observed in these WLMs reinforced with CNCs.

The present paper aims to study the temperature performance of WLMs by adding CNCs to a widely available and commonly used anionic surfactant, potassium oleate (PO). Dynamic and steady-shear rheological tests were conducted for the PO-CNC mixture and its individual components. It was observed that the addition of nanocrystals significantly increases the viscosity and storage modulus and improves their temperature stability. Moreover, CNCs cause heat-induced gelation of the PO-CNC mixture. The structural basis for this behavior was revealed by cryo-electron microscopy (cryo-TEM), which shows that WLMs and CNCs co-assemble into fibrillar-like aggregates that form a three-dimensional network. Using ITC, the forces driving PO-CNC aggregation were identified. By small-angle neutron scattering (SANS), it was shown that the nanostructure of PO-CNC aggregates is preserved during heating. These findings are significant for various practical applications of WLMs, particularly at elevated temperatures.

## 2. Results and Discussion

### 2.1. Behavior at Room Temperature

In the experiments, negatively charged carboxymethylated CNCs were used, with concentrations ranging from 0.5 to 8 wt%. At concentrations exceeding ca. 1 wt% [35,40], CNCs form a percolated network, where the rods preferentially touch each other by their hydrophobic ends [41,42]. These networks were suggested to be more effective than individual nanoparticles for reinforcement [13], since they provide a solid skeleton for the system.

Nanocrystals were added to the solutions containing a constant amount of surfactant PO (3 wt%) and two different concentrations of salt KCl (1.3 and 2.6 wt%). The salt screening of the electrostatic repulsion between the surfactant head groups favors the formation of longer WLMs [43]. This helps to regulate not only the length of the WLMs, but also their degree of entanglement. As a result, at 1.3 wt% KCl, the PO solution represents a dilute solution of rather short WLMs, whereas at 2.6 wt% KCl, long and highly entangled WLMs form. Below, these two cases will be considered separately. All the experiments were performed at pH = 10.5, where both micelles [44] and nanoparticles [35] are fully ionized and similarly charged. This ensures the colloidal stability of the PO-CNC suspensions, which do not phase separate for at least one month. No inhomogeneities were detected by optical microscopy as well (Figure S1).

#### 2.1.1. Rheological Properties of Dilute Solutions of Short WLMs After the Addition of CNCs

**Figure 1:**
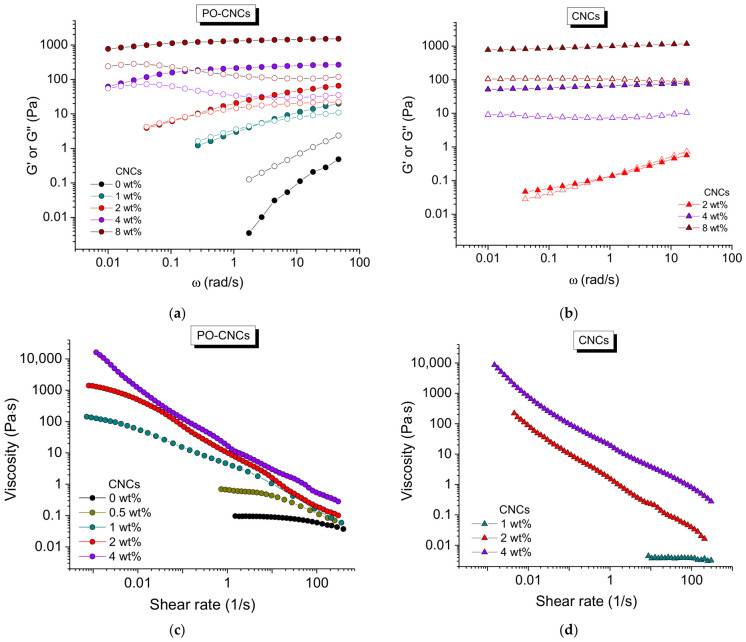
(**a**,**b**) Frequency dependences of storage $G^{\prime }$ (filled symbols) and loss $G^{\prime \prime }$(open symbols) moduli (**a**) for suspensions containing 3 wt% PO and different CNC concentrations and (**b**) for CNC suspensions without PO. (**c**,**d**) Steady-shear viscosity plots (**c**) for suspensions containing 3 wt% PO and different CNC concentrations and (**d**) for CNC suspensions without PO. Concentrations of CNCs are indicated in the figure. Solvent: 1.3 wt% KCl in water, pH 10.5. Temperature: 20 °C.

The frequency dependences of the storage $G^{\prime }\;$ and loss $G^{\prime \prime }$ moduli of 3 wt% PO solutions with 1.3 wt% KCl and different concentrations of added CNCs are presented in Figure 1a. It is seen that the PO solution without CNCs exhibits fluid-like behavior when the viscous response is greater than the elastic one $G^{\prime \prime }>G^{\prime }$ over the entire range of frequencies. When more than 1 wt% of CNCs is added, the PO-CNC system starts to behave like a viscoelastic liquid, when at high frequencies, the elastic behavior prevails ($G^{\prime }>G^{\prime \prime }$), whereas at low frequencies, the viscous behavior becomes dominant ($G^{\prime \prime }>G^{\prime }$). At CNC concentrations above 4 wt%, $G^{\prime }\;$ becomes higher than $G^{\prime \prime }\;\left(G^{\prime }>G^{\prime \prime }\right)$ at all the studied frequency range, which is characteristic of the viscoelastic solids. At these concentrations, the $G^{\prime }$(ω) curve shows a plateau (Figure 1a). Thus, CNCs induce a transformation of the fluid-like WLM solution into a gel-like state. Similar behavior was observed previously when various inorganic nanoparticles were added to dilute WLM solutions [11,18]. After adding 8 wt% of CNCs, the storage modulus of the PO-CNC gel becomes as high as 1480 Pa, which is ca. 3000 times greater than that for the initial PO solution.

**Figure 2:**
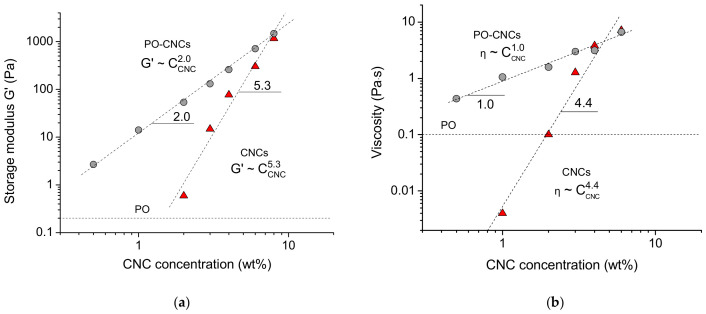
(**a**) Dependences of storage modulus $G^{\prime }$ at a frequency of 20 rad/s on CNC concentration for PO-CNC suspensions containing 3 wt% PO (gray circles) and for CNC suspensions without PO (red triangles). (**b**) Dependences of viscosity at a shear rate of 10 s^−1^ on CNC concentration for PO-CNC suspensions containing 3 wt% PO (gray circles) and for CNC suspensions without PO (red triangles). The data for PO solutions are indicated by horizontal dashed lines. Solvent: 1.3 wt% KCl in water, pH 10.5. Temperature: 20 °C.

Figure 2a shows that PO-CNC suspensions always have a higher storage modulus $G^{\prime }$ than CNC suspensions. This indicates that WLMs significantly contribute to the elasticity of the system, even though pure WLM solutions are fluid-like. Moreover, at 0.5–2.9 wt% CNCs, only the PO-CNC suspension demonstrates an elastic response, while neither pure CNC nor pure PO exhibits any elasticity. This suggests that they form a joint network in which subchains are composed of both WLMs and CNCs. As a result, a synergistic effect is observed. The $G^{\prime }$ values of PO-CNC suspensions significantly exceed the sum of the $G^{\prime }$ values of its components. For example, at 3 wt% CNCs, the storage modulus of the PO-CNC mixture is 135 Pa, whereas for pure CNCs it is 1.1 Pa and for pure PO it is only 0.2 Pa. Note that the storage modulus of the PO-CNC mixture scales with CNC concentration in a power-law relationship $G^{\prime }\;\sim {\;C}_{CNC}^{2.0}\;$ that is much weaker than that for a pure CNC suspension ($G^{\prime }\;\sim {\;C}_{CNC}^{5.3}$), but close to that usually observed for solutions of WLMs [45] and polymers [46]. The high exponent for a pure CNC suspension, already reported previously [35,40], was attributed [35] to the growth of long and thick bundles of nanocrystals at increasing CNC concentrations. This suggestion was supported by cryo-TEM and cryo-electron tomography data [39]. The smaller exponent observed in the PO-CNC mixture may testify that the CNC bundles are disrupted by the surfactant.

The flow curves for the same PO-CNC samples are shown in Figure 1c. Before the addition of CNCs, the PO solution has a low zero-shear viscosity of 0.1 Pa·s, indicating that the WLMs are rather short. These micelles demonstrate a shear-thinning behavior because of their alignment in the flow direction [47,48]. Starting from a CNC concentration of 1 wt%, the shape of the flow curve changes. Instead of one slope in the shear thinning region, it demonstrates several slopes typical for CNC suspensions (Figure 1d). The first slope at low shear rates is attributed to the orientation of domains composed of multiple nanocrystal particles in the direction of flow. At higher shear rates, these domains disintegrate into individual particles, and the final slope reflects the alignment of these individual CNC particles [49,50]. At CNC concentrations above 2 wt%, the zero-shear plateau observed in the PO solution disappears, and the flow curve becomes mostly determined by CNCs. When viscosity is defined by individual CNC particles (at high shear rates), it scales linearly with the CNC concentration $\eta \;\sim {\;C}_{CNC}^{1.0}$ (Figure 2b). Under the same conditions, pure CNC suspensions exhibit a much steeper increase in viscosity: $\eta \;\sim {\;C}_{CNC}^{4.4}$. These data suggest that surfactant affects significantly the flow behavior of CNCs, which is consistent with our suggestion about the formation of WLM–CNC aggregates.

Figure 1c shows that upon addition of CNCs, at a low shear rate of 0.001 s^−1^, the viscosity reaches 16,000 Pa·s, demonstrating an increase of more than five orders of magnitude compared to the PO solution. In contrast, at high shear rates (e.g., 100 s^−1^), the increase in viscosity caused by the nanoparticles is much smaller, by less than one order of magnitude. This greatly enhances the shear-thinning properties, which are crucial for various practical applications involving the pumping or injection of WLM solutions.

Thus, the addition of CNCs to a dilute solution of short WLMs induces a dramatic enhancement in rheological properties. A fluid-like system transforms into a viscoelastic solid, with a storage modulus exceeding the loss modulus over the entire range of frequencies studied. The storage modulus rises by up to four orders of magnitude. Simultaneously, the viscosity increases by more than five orders of magnitude at low shear rates and by up to one order of magnitude at high shear rates. The observed effect of CNCs on the storage modulus and viscosity is greater than that of previously studied WLM-CNC systems [28,29].

#### 2.1.2. Rheological Properties of Semi-Dilute Solutions of Long WLMs After the Addition of CNCs

**Figure 3:**
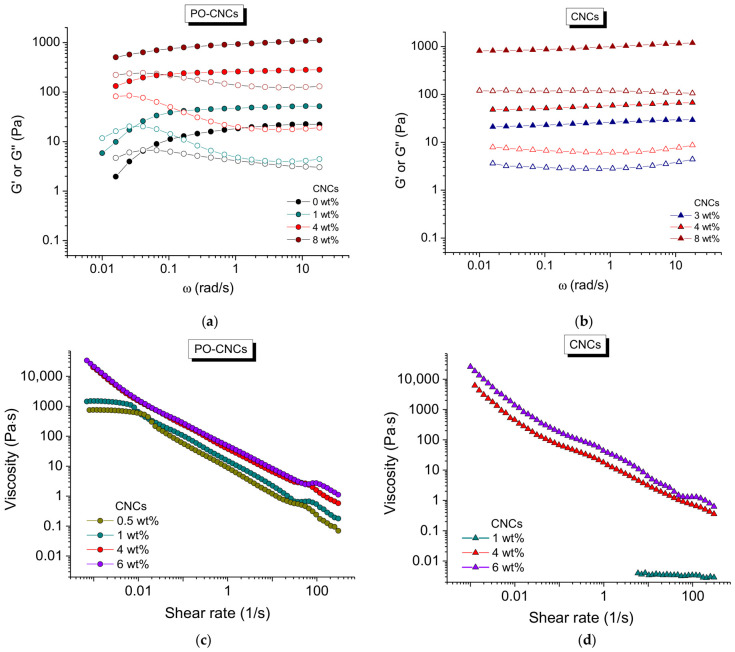
(**a**,**b**) Frequency dependences of storage $G^{\prime }$ (filled symbols) and loss $G^{\prime \prime }$ (open symbols) moduli (**a**) for suspensions containing 3 wt% PO and different CNC concentrations and (**b**) for CNC suspensions without PO. Data for the pure PO solution were taken from [52]. (**c**,**d**) Steady-shear viscosity plots (**c**) for suspensions containing 3 wt% PO and different CNC concentrations and (**d**) for CNC suspensions without PO. Concentrations of CNCs are indicated in the figure. Solvent: 2.6 wt % KCl in water, pH 10.5. Temperature: 20 °C.

**Figure 4:**
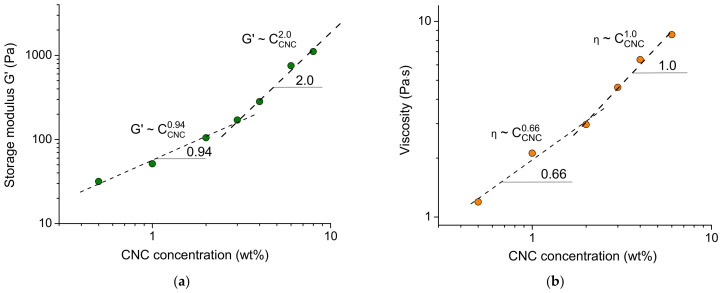
(**a**) Dependence of storage modulus $G^{\prime }$ at a frequency of 20 rad/s on CNC concentration for PO-CNC suspensions containing 3 wt% PO. (**b**) Dependence of viscosity at a shear rate of 10 s^−1^ on CNC concentration for PO-CNC suspensions containing 3 wt% PO. Solvent: 2.6 wt% KCl in water, pH 10.5. Temperature: 20 °C.

The frequency dependences of the storage $G^{\prime }$ and loss $G^{\prime \prime }$ moduli for semi-dilute solutions of long entangled WLMs containing 3 wt% PO and 2.6 wt% KCl are shown in Figure 3a. Before the addition of CNCs, the solution exhibits viscoelastic properties typical of entangled WLMs [51] and reported previously [52]. At low shear rates, the viscous response prevails $\;{(G}^{\prime \prime }>\;G^{\prime }),\;$ while at high shear rates, the elastic response dominates ($G^{\prime }>$ $G^{\prime \prime }),$ and there is a plateau on the $G^{\prime }$(ω) curve. The addition of the CNCs results in the growth of the plateau modulus $G_{0}$ by up to two orders of magnitude. With increasing CNC concentration, the range of frequencies corresponding to the predominant elastic response ($G^{\prime }>$ $G^{\prime \prime }$) becomes wider. Finally, at 4 wt% CNCs, the intersection between the $G^{\prime }$(ω) and $G^{\prime \prime }$(ω) curves disappears, indicating a viscoelastic solid state of the PO-CNC mixture. This occurs at the same concentration of nanocrystals as at low salt content, when the WLMs are much shorter, which suggests that the transition to a viscoelastic solid is mainly determined by the contribution of the CNCs. Figure 4a shows the evolution of storage modulus with CNC concentration. In the log-log plot, the curve has two distinct slopes: 0.94 for CNC concentrations below 3 wt% and 2.0 for higher CNC concentrations. The second slope is the same as that for short WLMs (Figure 2a). The first slope, which is observed only for long WLMs, is likely due to the contribution of entanglements between WLMs in the $G^{\prime }$ value under conditions where the influence of CNCs is less than that of the WLMs.

The effect of CNCs on the flow curves is illustrated in Figure 3c. At low CNC content, one can observe the behavior typical for WLMs, characterized by a zero-shear plateau and shear-thinning at high frequencies due to the alignment of the micelles [47,48]. When the CNC content becomes higher than 4 wt%, the Newtonian plateau at low shear rates disappears, while several slopes appear in the shear-thinning region, which is typical for CNC suspensions [49,50]. At low concentrations of CNCs, a significant contribution of PO to viscosity is observed, while at $C_{CNC}>$ 4 wt%, the viscous behavior of the mixture is close to that of the pure CNC suspension. The concentration dependence of viscosity (Figure 4b) in a log-log plot has two slopes: 0.66 and 1.0. The second slope, seen at high CNC concentrations, is similar to that for short WLMs (Figure 2b), while the first slope is slightly smaller. This suggests that at low CNC concentrations, the relative impact of CNCs in the rheological properties decreases with increasing length of WLMs.

Thus, when CNCs are added to semi-dilute solutions of WLMs, both the storage modulus and viscosity increase by up to two orders of magnitude, resulting in a transition of the viscoelastic fluid into a viscoelastic solid. At low concentrations of PO, WLMs contribute significantly to the viscoelasticity of the mixture, but when more than 4 wt% CNCs are added, the nanoparticles start to play a dominant role in the rheological properties of the mixture, providing a viscoelastic solid state.

#### 2.1.3. Interaction Between Surfactant and CNCs

The observed enhancement in the rheological properties of WLMs by added CNCs, especially under conditions where both WLMs and CNCs are unable to form their own networks in the whole volume of the system, can be explained by the attractive interactions between them, which lead to the formation of a joint network structure. Since the micelles and the nanoparticles in the present system have similar charges, it is expected that the attractive forces between them are due to hydrogen bonding and hydrophobic interactions. To reveal the prevailing interactions between PO micelles and CNCs, ITC studies were performed.

**Figure 5:**
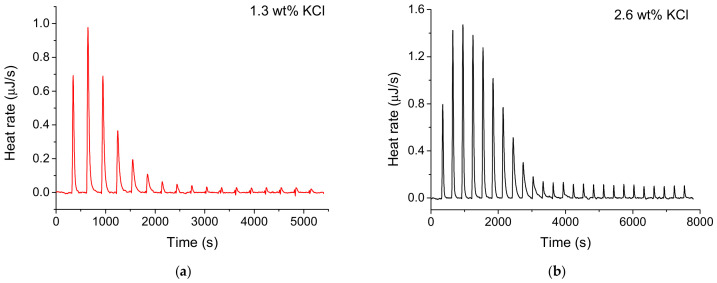
The thermograms of isothermal titration obtained by the gradual injection of 2 µL of 0.05 wt% aqueous solution of PO into 170 µL of 0.3 wt% suspensions of CNCs in the presence of 1.3 (**a**) and 2.6 wt% (**b**) of KCl at pH 10.5. Temperature: 20 °C.

In Figure 5, the thermograms of the titration of 0.3 wt% CNC suspensions with 0.05 wt% PO solutions containing 1.3 and 2.6 wt% KCl are presented. The concentration of PO in the titrant was kept significantly lower than the CMC in order to avoid the destruction of PO micelles during dilution. The measured heat rates of PO injections into water (Figures S2a and S3a) were 1–2 orders of magnitude lower compared to those obtained upon addition of PO in CNC suspensions at the same salinities (Figure 5). Moreover, they were of the same order as the heat rates in the corresponding thermograms of titration with aqueous solutions of KCl without PO (Figures S2b and S3b). Thus, Figure 5 contains data on the energy release during the interaction of PO with CNCs, and the possible thermodynamic effect of PO micelle formation can be neglected.

**Figure 6:**
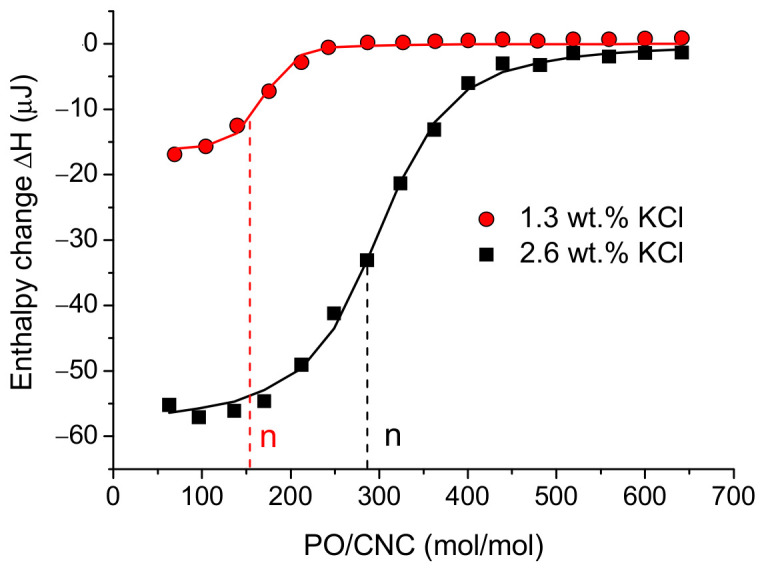
The thermodynamic profiles of isothermal titration of PO into suspensions of CNCs in the presence of 1.3 and 2.6 wt% of KCl at pH 10.5. The solid lines are fittings of the profiles using an independent binding site interaction model. The dashed lines indicate the corresponding stoichiometric numbers *n*. Temperature: 20 °C.

The thermograms (Figure 5) indicate that the binding of the PO surfactant to the CNCs is exothermic. The corresponding thermodynamic profiles were obtained after integration of the thermograms (Figure 6). The thermodynamic parameters of the process obtained by fitting the profiles (Figure 6) using an independent binding site interaction model are summarized in Table 1. The negative sign of the Gibbs free energy change Δ*G* indicates the spontaneous binding at both studied concentrations of KCl [53]. The negative values of enthalpy changes Δ*H* and the positive values of entropy changes Δ*S* (Table 1) suggest that the PO-CNC binding is governed by both the gain in enthalpy and entropy: Δ*G* = Δ*H* − TΔ*S* < 0. Such behavior is usually observed in the case of hydrogen bonding [54]. Indeed, the formation of hydrogen bonds between the carboxylate groups of the surfactant and the -OH groups of CNCs has recently been confirmed through the displacement of corresponding absorption peaks in FTIR spectra [28]. Similar FTIR spectroscopy results were also obtained for sodium oleate WLMs interacting with cellulose nanofibrils [27,30]. The increase in entropy can be due to the release of water molecules surrounding the interacting PO and CNC species [55]. It may occur during hydrogen bonding [54] or hydrophobic interactions [56]. In the last case, the PO adsorption on CNC aims to reduce the thermodynamically unfavorable contact of alkyl tails of surfactant and hydrophobic domains of cellulose with water.

Table 1 allows for comparing the thermodynamic parameters of PO-CNC interactions at different salt contents, affecting the screening of the electrostatic repulsion between similarly charged interacting species. One can see that with increasing salt concentration, the gain in enthalpy becomes more significant, and the stoichiometric ratio *n* (the number of surfactant molecules per nanocrystal) doubles (Table 1). This suggests that stronger screening of electrostatic repulsion enhances attractive forces, such as hydrogen bonding, which favor PO-CNC association. This is in agreement with the Derjaguin–Landau–Verwey–Overbeek (DLVO) theory [57]. At the same time, the change in entropy becomes less significant as the salt content increases (Table 1), as expected, since the presence of more salt ions, which contribute significantly to overall entropy, reduces the relative contribution to entropy provided by the release of water molecules from interacting species. At both salt contents, the saturation of CNC with PO occurs simultaneously with the enthalpy change (Δ*H*) reaching zero (Figure 6), indicating that hydrogen bonding, which is responsible for negative values of Δ*H*, provides a dominant contribution to the binding of PO to CNC.

Thus, the attraction between the WLMs of PO and the CNCs seems to be mainly driven by hydrogen bonding, but hydrophobic interactions may also be involved.

#### 2.1.4. Structure and Morphology

**Figure 7:**
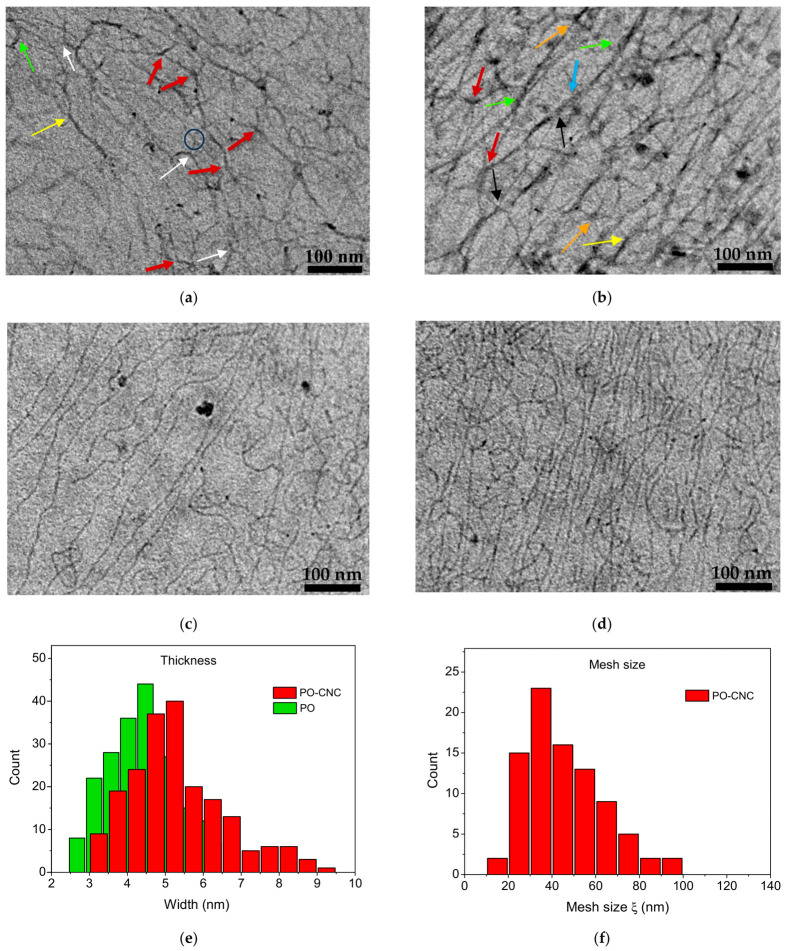
(**a**,**b**) Cryo-TEM images of suspensions containing 3 wt% PO and 2 wt% (**a**) or 0.5 wt% (**b**) CNCs. Kinks are marked by red arrows. CNCs wrapped with micelles are marked by green arrows; WLMs lying along the CNCs are marked by blue arrow. Crosslinks formed by three CNCs are marked by white arrows; crosslinks formed by more than three CNCs are marked by orange arrows. Black arrows show a long WLM connecting two fibrillar-like aggregates. Links between nanocrystals and WLM ends are marked by yellow arrows. Entanglement between WLMs is marked by a circle. (**c**,**d**) Cryo-TEM images of 3 wt% PO solutions. (**e**,**f**) Histograms of the distribution of the chain thickness (**e**) and mesh size (**f**) in suspensions containing 3 wt% PO and 2 wt% CNCs (red) and 3 wt% PO solutions (green). Solvent: 1.3 wt % KCl in water, pH 10.5.

The structure of PO-CNC suspensions was studied using cryo-TEM. Typical micrographs of PO-CNC suspensions and PO solutions without CNCs are shown in Figure 7a,b and Figure 7c,d, respectively. As can be seen from the micrographs, PO-CNC suspensions contain much thicker and darker chain-like structures compared to WLMs. These fibrillar-like structures are micrometer-long and contain many straight fragments that are linked together at their ends. Sometimes, kinks can be seen at the points where the straight fragments are connected (red arrows). These straight fragments represent individual nanocrystals. Almost no laterally aggregated nanocrystals (bundles) typical for CNC suspensions [39,40] can be found. In addition to straight fragments, the fibrillar-like structures contain some flexible elements that represent WLMs. One can detect many links between the WLMs and nanocrystals. Most of these links are formed via the end parts of nanocrystals (yellow arrows). However, in some cases, micelles can wrap around nanocrystals (green arrows) or lie parallel to them (blue arrow). The histogram (Figure 7e) shows that the average thickness of fibrillar-like aggregates is greater than that of WLMs. This can be attributed to the contribution of CNCs, which have a larger thickness (ca. 6 nm) compared to WLMs (ca. 4 nm). Additionally, in the PO-CNC suspension, a peak at ca. 8 nm appears, which may be due to CNCs decorated with micelles when they wrap around or lie parallel to nanocrystals. 

To the best of our knowledge, this is the first observation of fibrillar-like aggregates composed of both WLMs and CNCs. So far, three WLM–organic nanocrystal systems have been studied [13,28,29]. In an oppositely charged WLM–CNC system [28], cryo-TEM shows some CNCs dispersed in a network of entangled WLMs with only a few contacts between WLMs and CNCs. Visually, the structure differs from the fibrillar-like aggregates we observed. In a similarly charged WLM–nanocrystal system [13], two separate networks form: one composed of entangled WLMs and the other of percolated nanocrystals. No structural data were provided for the third system [29].

Figure 7a,b shows that fibrillar-like aggregates are crosslinked with each other, forming a network. Both nanocrystals and WLMs participate in crosslinking, forming three types of crosslinks. The first type is CNC-to-CNC crosslinks. Most frequently, they involve three CNCs (white arrows), located at an angle of 120°, possibly due to repulsion of charged groups on their side surfaces. Indeed, according to the analysis of wide-angle X-ray scattering data [41], only the side surfaces of CNCs bear charged groups, while the edge planes do not. Sometimes, four to five nanocrystals join together in a single crosslink (orange arrows). In all the cases, the CNCs touch each other at their ends, which are uncharged [41]. The second type of crosslinks is between CNCs and WLMs. For instance, black arrows show a long WLM connecting two fibrillar-like aggregates. WLMs can interact with the ends of CNCs (yellow arrows) or with the middle parts of CNCs, either wrapping around them (green arrows) or lying along them (blue arrow). One can speculate that when the ends of CNCs participate in interactions, hydrophobic interactions are more likely to occur, as the ends have a hydrophobic surface ((200) plane edge), which is mainly composed of CH groups [41,42]. In the case of WLMs wrapping around or lying along the nanocrystals, the hydrogen bonds are expected to dominate, as the OH groups are mainly located on the lattice planes (110), (1$\bar{1}$0) [42,58]. The third type of crosslinks represents entanglements between WLMs (marked by a circle). Note that WLM-to-WLM junctions were not found, probably because the WLMs under study are linear and do not branch. On the micrograph, one can distinguish the entanglements, where two WLMs cross each other (X-shaped connections), from the branching points, where three WLM fragments share one junction (Y-shaped connections) [59]. At 2 wt% CNCs, the PO-CNC suspensions have a structure similar to that at 0.5 wt% CNC, as shown in the cryo-TEM images (Figure 7a,b and Figure S4). Figure 7f shows the histogram of the distribution of mesh size ξ in the PO-CNC network containing 2 wt% CNCs (Figure S4b). In the histogram, the maximum is observed at 35 nm, which is close to the mesh size of 38 ± 5 nm obtained from the plateau value of the corresponding $G^{\prime }$(ω) curve [52]. 

**Figure 8:**
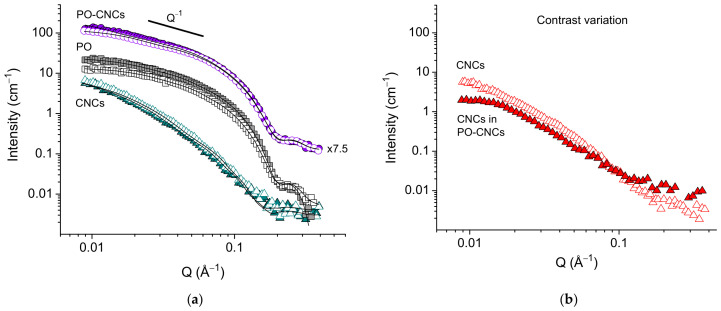
(**a**) SANS profiles for suspension containing 3 wt% PO and 2 wt% CNCs (circles) and its components: 3 wt% solution of PO (squares) and 2 wt% suspension of CNCs (triangles) in the presence of 1.3 wt% (open symbols) and 2.6 wt% KCl (filled symbols) in D_2_O. The PO-CNC curve is shifted for a better representation (×7.5); the other curves are in absolute values. Thin lines are best fits of the scattering data by a model of cylinders for PO-CNC and PO, and a model of parallelepiped for CNCs. The bold line shows the Q^−1^ slope characteristic for a form factor of a cylinder. (**b**) SANS profiles with contrast variation for suspension containing 3 wt% PO, 2 wt% CNCs, and 2.6 wt% KCl in the mixture 10% D_2_O/90% H_2_O matching PO (filled symbols) and for suspension containing 2 wt% CNCs and 2.6 wt% KCl without PO (open symbols) in D_2_O. Temperature: 25 °C.

The structure of PO-CNC suspensions on a 1–70 nm scale was studied by SANS. At 2 wt% CNCs, these suspensions demonstrate the typical features of WLM chains (Figure 8a). In the intermediate range of wave vectors *Q*, the scattering curve has a slope of ca. −1, consistent with the cylindrical shape of the scattering objects. At high *Q*, the scattering can be fitted by a form factor of a solid cylinder, yielding a cross-sectional radius of 1.8 nm, which is the same as in a pure PO solution. This suggests that the micelle structure remains intact at the nanoscale upon addition of CNCs. When comparing the data at two salt concentrations (Figure 8a), one can see that in pure PO at smaller salt content, the scattering decreases at low *Q*, indicating that the micelles become shorter. In PO-CNC suspension, the decrease in the scattering intensity is much less pronounced. This can be due to the contribution from nanocrystals at low *Q* values.

At high and intermediate *Q* values, the contribution of nanocrystals to the scattering of the PO-CNC system is small because of their low contrast. In order to reveal the structure of the nanocrystals in the PO-CNC suspension, a contrast variation that matches the PO scattering was used (Figure 8b). The SANS profiles of CNCs obtained in this way were fitted with a form factor of a parallelepiped, which has been shown to best describe the scattering from CNCs [60,61]. The fitting parameters provide the following dimensions for the parallelepiped at 1.3 wt% KCl: 112 nm in length, 16 nm in width, and 3.6 nm in thickness. The thickness of 3.6 nm is close to the thickness of individual CNCs, while the length of 112 nm is only slightly longer than the length of CNCs (90 nm) determined from cryo-TEM data [39] on the same CNC samples. Meanwhile, the width of the parallelepiped is four times larger than its thickness. This can be attributed to either the characteristic sheet-like configuration of individual nanocrystals [60] or the lateral aggregation of several nanocrystals [62]. At 2.6 wt% KCl, the width and thickness remain almost the same, but the length of nanocrystals slightly increases from 112 to 132 nm, which may indicate that salt favors the end-to-end aggregation of CNCs. Note that at 20 °C, the length of CNCs is 112 nm (at 2.6 wt% KCl), according to SANS [52]. Therefore, not only the addition of more salt, but also heating from 20 to 25 °C promotes the aggregation of CNCs via their hydrophobic ends. The size of CNCs in the PO-CNC suspension is the same as in the CNC suspension (without PO). Thus, in PO-CNC suspensions, SANS data reveal the presence of long, cylindrical scattering objects, in which the local structure of both WLMs and CNCs is preserved at the nanoscale. Higher salt concentration promotes the elongation of WLMs and the end-to-end aggregation of CNCs.

Overall, structural studies evidence that WLMs and CNCs co-assemble into long, fibrillar-like aggregates crosslinked with each other into a network, which provides enhanced rheological properties that are far superior to those of WLMs alone.

### 2.2. Behavior upon Heating

#### 2.2.1. Rheological Properties

The effect of temperature on the rheological properties was studied on an example of long WLMs (at 2.6 wt% KCl), which are expected to maintain viscoelasticity over a wider range of temperatures. The experiments were performed at 3 wt% PO and two different concentrations of CNCs: 2 and 4 wt%. At 2 wt% of CNCs, WLMs still significantly affect the rheological properties, while at 4 wt% of CNCs, the contribution of nanocrystals becomes dominant.

**Figure 9:**
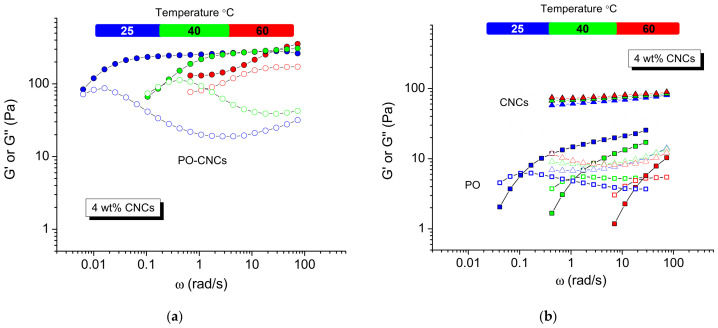

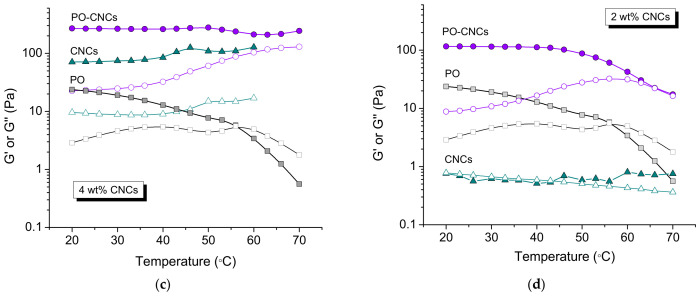
(**a**,**b**) Frequency dependences of storage $G^{\prime }$ (filled symbols) and loss $G^{\prime \prime }$ (open symbols) moduli for (**a**) suspension containing 3 wt% PO and 4 wt% CNCs (circles) and (**b**) its components: 3 wt% solution of PO (squares) and 4 wt% suspension of CNCs (triangles) at 25 (blue), 40 (green) and 60 °C (red). (**c**) Variation in storage $G^{\prime }$ (filled symbols) and loss $G^{\prime \prime }$ (open symbols) moduli at a frequency of 20 rad/s with temperature for suspension containing 3 wt% PO and 4 wt% CNCs (circles) and its components: 3 wt% solution of PO (squares) and 4 wt% suspension of CNCs (triangles). (**d**) Variation in storage $G^{\prime }$ (filled symbols) and loss $G^{\prime \prime }$ (open symbols) moduli at a frequency of 20 rad/s with temperature for suspension containing 3 wt% PO and 2 wt% CNCs (circles) and its components: 3 wt% solution of PO (squares) and 2 wt% suspension of CNCs (triangles). Solvent: 2.6 wt% KCl in water, pH 10.5.

Let us first consider the data for samples with 4 wt% CNCs. Figure 9 shows the frequency dependences of the storage and loss moduli at temperatures varying from 25 to 60 °C. The curves for pure surfactant solution (Figure 9b) demonstrate that the cross-over point of $G^{\prime }(\omega )$ and $G^{\prime \prime }(\omega )$ shifts to higher frequencies, and the plateau of storage modulus $G_{0}$ disappears upon heating. Such behavior, previously reported in many papers [2,8], was attributed to the shortening of micellar chains. The equilibrium micelle length is determined by a balance between the enthalpic cost of forming hemispherical endcaps, which lack favorable tail-tail interactions, and the entropic penalty of maintaining long micelles [63]. As temperature increases, the entropic penalty becomes more significant, leading to a shortening of the micellar chains. The plateau of storage modulus $G_{0}\;$ disappears when the micelles become too short to form entanglements. In contrast, the CNC network exhibits some strengthening at higher temperatures, which is manifested in a slight increase in the storage modulus $\;G^{\prime }$ (Figure 9b). The strengthening of the CNC network can be due to the enhancement in hydrophobic interactions between the nanocrystals [64,65,66].

Figure 9 evidences that the PO-CNC mixture demonstrates better temperature stability of its viscoelastic properties compared to the PO solution. When the mixture is heated from 20 to 40 °C, the plateau modulus remains unchanged, in contrast to a pure PO solution, where it decreases (Figure 9a,b). At the same time, the point of intersection between $G^{\prime }(\omega )$ and $G^{\prime \prime }\left(\omega \right)$ shifts to higher frequencies, indicating a decrease in the terminal relaxation time (Figure 9a). This suggests that the micelles shorten, but the average length of most of them should remain greater than the entanglement length, as the plateau modulus does not change. Upon further heating to 60 °C, the $G^{\prime }$ values become even slightly greater at the highest frequencies (Figure 9a). Simultaneously, the crossover point disappears, and $G^{\prime }$ becomes always higher than $G^{\prime \prime }$ suggesting an increased contribution of CNCs to the relaxation process. Thus, in the PO-CNC system, at 60 °C, the elastic response becomes dominant ($G^{\prime }>\;G^{\prime \prime }$) throughout the entire studied frequency range, indicating the transition of viscoelastic fluid to a viscoelastic solid, which is induced by added nanoparticles. Note that for a pure CNC network, $G^{\prime }$ is one order of magnitude higher than $G^{\prime \prime }$ and only slightly dependent on frequency (Figure 9b). At the same time, for a PO-CNC network, $G^{\prime }$ is only up to two-fold higher than $G^{\prime \prime }$ and strongly frequency dependent (Figure 9a). This may be due to the presence of WLM fragments in the fibrillar-like aggregates forming the subchains. The dynamics of these fragments, which can break and reform anywhere along their length, are faster than the dynamics of a pure CNC network, where breaking and reformation only occur at CNC-CNC contacts. At the same time, the absolute $G^{\prime }$ values of the mixed PO-CNC network are up to four times higher compared to a pure CNC network due to the contribution of WLMs, which provide more material for the construction of the network.

Figure 9c shows the effect of temperature on the storage $G^{\prime }$ and loss $G^{\prime \prime }$ moduli at a fixed frequency of 20 rad/s. For the PO solution, the storage modulus decreases significantly upon heating. By contrast, for the CNC suspension, one can observe a small increase in the storage modulus with temperature, which may be due to the enhancement in hydrophobic interactions between the nanoparticles. As to the PO-CNC mixture, its storage modulus $G^{\prime }\;$ remains almost unchanged over the entire studied temperature range (20–70 °C), demonstrating excellent temperature tolerance. At the same time, the loss modulus increases with heating, which may be due to the viscous contribution from WLMs becoming shorter at higher temperatures.

When considering PO-CNC suspension at a lower CNC content (2 wt%), one can see (Figure S5 and Figure 9d) that its storage modulus does not significantly change until ca. 50 °C. Upon further heating, it decreases, but this decrease is less significant compared to pure WLMs. For instance, upon heating from 20 to 60 °C, the storage modulus in the presence of CNCs decreases by only a factor of 8.5, whereas in the absence of CNCs it drops by a factor of 130. So, even at rather low CNC content, the elastic properties of the PO-CNC mixture are more temperature-stable than those of pure PO due to added nanoparticles. At 2 wt% CNC, heat-induced gelation is also observed (Figure S5a), as in the case of the PO-CNC suspension with 4 wt% CNCs. However, the critical gel temperature at 2 wt% CNC is higher (55 °C) than that at 4 wt% CNC (45 °C), as can be seen from Figure S6, based on the temperatures at which the crossover of $G^{\prime }(\omega )$ and $G^{\prime \prime }\left(\omega \right)$ curves disappears.

The impact of the CNCs on the PO-CNC storage modulus at different temperatures can be clearly seen in Figure S7, which shows the ratio of the $G^{\prime }$ values for PO solutions with and without CNCs as a function of temperature. One can see that the contribution of CNCs to the storage modulus increases tremendously upon heating above 60 °C. This occurs because the entanglements between WLMs disappear and the CNC network becomes the main contributor to elasticity. The increase in the ratio of the $G^{\prime }$ values for PO solutions with and without CNCs is much more pronounced at higher CNC content. At 70 °C, it reaches 30 and 430 at 2 and 4 wt% of CNCs, respectively (Figure S7).

**Figure 10:**
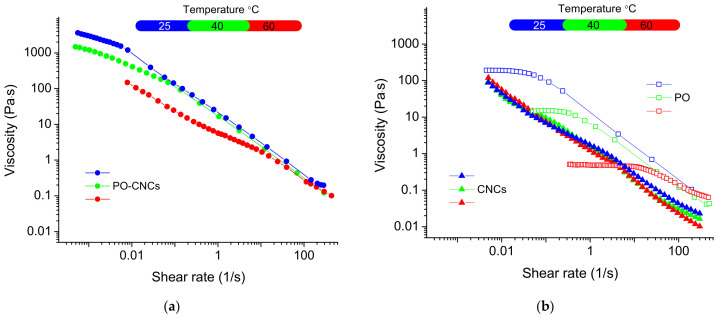
Flow curves for (**a**) suspension containing 3 wt% PO and 2 wt% CNCs (circles) and (**b**) its components: 3 wt% solution of PO (squares) and 2 wt% suspension of CNCs (triangles) at 25 (blue), 40 (green) and 60 °C (red). Solvent: 2.6 wt.% KCl in water, pH 10.5.

Figure 10 shows the flow curves at different temperatures. It is seen that the viscosity of pure CNC suspension does not change significantly upon heating (Figure 10b). In contrast, for PO solutions, the viscosity decreases upon heating, both with (Figure 10a) and without (Figure 10b) nanocrystals. However, in the presence of CNCs, this decrease is much less pronounced. For instance, upon heating from 20 to 40 °C, the zero-shear viscosity of the PO-CNC mixture with 2 wt% CNCs shows only a three-fold decrease, instead of a 30-fold decrease, when CNCs are not added. At 60 °C, the flow curve for the PO-CNC mixture resembles that of pure CNC suspension (Figure 10), suggesting that CNCs play the major role in determining the viscosity under these conditions. Consequently, CNCs are responsible for the higher viscosity of the PO-CNC mixture compared to pure PO solution at elevated temperatures.

Thus, CNCs impart better temperature stability to WLM solutions and even induce heat-induced gelation when the CNC network starts to play a major role in the rheological behavior of the PO-CNC mixture.

#### 2.2.2. Structure and Interactions That Stabilize It

The study of the structural features behind the increased temperature tolerance of WLM solutions with added organic nanocrystals and heat-induced gelation is very important, as it can help to develop new systems with these properties. First, optical microscopy studies were performed at both room and elevated temperatures (Figure S1). These studies revealed that the PO-CNC suspension remained homogeneous during heating, with no microphase separation observed. This finding contrasts with data obtained for another system comprising similarly charged WLMs and nanocrystals that also exhibits heat-induced gelation [13]. In that system, the increased temperature tolerance and heat-induced gelation were shown to be due to the formation of a nanocrystal network within a network of entangled WLMs and a microphase separation, with the formation of bicontinuous nanocrystal-rich and nanocrystal-poor phases, which effectively concentrated nanocrystals and WLMs locally, strengthening both the nanocrystal and micellar networks. In the present PO-CNC system, both WLMs and CNCs assemble together into common fibrillar-like aggregates that form a single network rather than two separate networks, as reported in the referenced paper [13]. These findings suggest that the reasons for heat-induced gelation in the current system may differ from those reported in the cited study [13], even though both systems are composed of similarly charged WLMs and nanocrystals.

**Figure 11:**
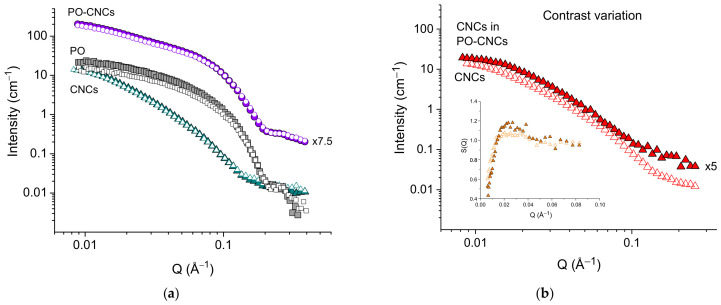
(**a**) SANS profiles for suspension containing 3 wt% PO and 4 wt% CNCs (circles) and its components: 3 wt% solution of PO (squares) and 4 wt% suspension of CNCs (triangles) in the presence of 2.6 wt% KCl in D_2_O at 25 °C (filled symbols) and 60 °C (open symbols). The PO-CNC curve is shifted for a better representation (×7.5); the other curves are in absolute values. (**b**) SANS profiles with contrast variation for suspension containing 3 wt% PO, 4 wt% CNCs, and 2.6 wt% KCl in the mixture 10% D_2_O/90% H_2_O matching PO (filled symbols) and for suspension containing 4 wt% CNCs and 2.6 wt% KCl without PO (open symbols) in D_2_O at 60 °C. The curve for CNCs in PO-CNCs is shifted for a better representation (×5), and another curve is in absolute values. Inset: Structure factor for the same suspensions.

The influence of heating on the structure of PO-CNC suspensions on a 1–70 nm scale was studied by SANS on an example of suspensions containing 4 wt% CNCs. It was observed that PO-CNC curves at 25 and 60 °C are almost identical (Figure 11), suggesting that the local structure of the micelles is preserved during heating. At the same time, the scattering of pure PO solution decreases at low *Q*, which is an indication of a shortening of the WLMs. In the PO-CNC system, this decrease could not be seen because of the contribution of CNCs to this part of the scattering curve.

Figure 11b shows the SANS curves of CNCs in the PO-CNC suspension obtained by contrast variation matching the scattering of PO. Fitting of the scattering curves with a form factor of a parallelepiped gives the following dimensions for the parallelepiped: length of 107 nm, width of 15 nm, and thickness of 3.8 nm. The fitting parameters are almost the same at both 25 and 60 °C. This means that the nanoscale structure of CNCs remains intact during heating. To estimate the spatial arrangement and interactions between CNC particles in the PO-CNC suspension, the structure factor S(*Q*) was evaluated. This was done by dividing the total scattering intensity by the form factor F(*Q*), which was taken from scattering data for a dilute 0.5 wt% CNC suspension (without PO) (Figure S8), in accordance with the formula [66]: S(*Q*) = I(*Q*)/F(*Q*). The structure factor thus obtained is presented in Figure 11 (inset). One can see that the interaction between the CNC particles in the PO-CNC suspension is stronger than in the CNC suspension. This indicates a decrease in the correlation distance between the CNCs when they are included in fibrillar-like aggregates, compared to pure CNC suspension [67]. Note that the structure factor has not changed after heating, demonstrating the temperature tolerance of the PO-CNC fibrillar-like aggregates. This finding can explain the reason for the temperature stability of the storage modulus of the WLM solutions containing CNCs (Figure 9).

To reveal the driving forces behind the stabilization of PO-CNC aggregates during heating, ITC was used. Figure S9a displays the raw thermogram of the isothermal titration of CNCs with PO in the presence of 2.6 wt.% KCl at 60 °C. The corresponding thermodynamic profile (Figure S9b) was obtained by integrating the thermogram after subtracting the peaks of the PO titration into an aqueous solution of KCl at the same temperature (Figure S10). This allows us to reveal the thermodynamic effect of the PO-CNC interaction, while excluding the process of micelle breakdown. Fitting the profile (Figure S9b) using an independent binding site interaction model gives the following thermodynamic parameters of PO-CNC interaction: the change in Gibbs free energy Δ*G* = −27 kJ/mol, the change in enthalpy ∆*H* = 30 kJ/mol, the change in entropy ∆*S* = 172 J/mol∙K. They show that at 60 °C, the enthalpy of PO-CNC interaction is positive, in contrast to 20 °C. This suggests that hydrogen bonding, which was the main driving force behind the PO-CNC interactions at room temperature, has lost its dominant role. Instead, the negative change in Gibbs free energy Δ*G* is now determined primarily by the entropy gain, which is likely due to the release of structured water molecules around the interacting species. Such behavior is typical for hydrophobic interactions that were shown [68,69] to be entropy-driven. Therefore, the ITC data evidence that at 60 °C, hydrophobic interactions play a leading role in PO-CNC aggregation. The change in the main driving force for the PO-CNC interactions from hydrogen bonding to hydrophobic interactions when heating can be attributed to two reasons: (i) an enhanced breaking of hydrogen bonds due to the increased thermal motion at higher temperatures [70,71,72] and (ii) a greater contribution of hydrophobic interactions at higher temperatures due to their entropic origin [71].

Thus, PO-CNC suspensions exhibit remarkable temperature tolerance due to the stability of the fibrillar-like PO-CNC aggregates, which is provided by hydrophobic interactions.

## 3. Conclusions

In this paper, the effect of the CNCs on the rheological properties of WLM solutions and their temperature stability was studied. It was shown that when CNCs are added to dilute solutions of short WLMs, the liquid-like solution first transforms into a viscoelastic fluid (at 1 wt% of CNCs) and then into a viscoelastic solid (at 4 wt% of CNCs). During this process, the storage modulus increases by up to four orders of magnitude. Simultaneously, the viscosity increases by more than five orders of magnitude at low shear rates and by up to one order of magnitude at high shear rates, making the shear-thinning properties much more pronounced. The observed effect of CNCs on the storage modulus and viscosity is greater than that in any previously studied WLM-CNC system [28,29].

When CNCs are added to semi-dilute solutions of long WLMs, the system, which initially represents a viscoelastic fluid, transforms into a viscoelastic solid when the amount of added CNCs exceeds 4 wt%. In this case, the PO-CNC suspension also exhibits superior viscoelastic properties compared to its individual components. The elastic modulus at the plateau and the apparent viscosity both increase by up to two orders of magnitude when the CNCs are added.

CNCs increase the temperature stability of the rheological properties of WLM solutions. The storage modulus of the PO-CNC system keeps a constant value upon heating from 20 to 70 °C, while for the pure PO solution, it decreases by a factor of 40. At increasing temperature, when the CNC network starts to play a major role in the rheological behavior of the PO-CNC mixture, a heat-induced gelation is observed. The critical gel temperature decreases from 55 to 45 °C with increasing content of CNCs in the PO-CNC mixture from 2 to 4 wt%.

The enhancement in the rheological properties of WLMs by nanocrystals was attributed to the formation of a joint network, in which the subchains represent the fibrillar-like aggregates formed by both WLMs and CNCs. Cryo-TEM data confirmed the suggested structures of the PO-CNC networks. The ITC results revealed that at room temperature, the dominant type of interactions between PO and CNCs responsible for strengthening the network is hydrogen bonding, while at elevated temperature (60 °C), the hydrophobic interactions prevail. SANS data showed that the local structure of both WLMs and CNCs at the nanoscale remained unchanged in the mixed systems, while the correlation distance between the CNCs, when they are included in fibrillar-like aggregates, is decreased compared to pure CNC suspension.

Improved temperature stability of WLMs together with enhanced rheological properties is highly required for their numerous industrial applications, particularly for their use as thickeners in fracturing fluids for oil recovery [13,28]. This would expand the range of temperature conditions in which WLMs can be effectively utilized. In addition, the enhanced shear thinning properties of WLM-CNC suspensions compared to pure WLMs would also be beneficial for this application. Indeed, during pumping in a well, which proceeds at high shear rates, the liquid will flow easily due to its rather low viscosity. This will reduce frictional losses and the power needed to operate the pumps. But once the liquid is left to rest inside the fracture, its viscosity will spontaneously increase by four orders of magnitude, providing excellent thickening ability.

## 4. Materials and Methods

### 4.1. Materials

PO (purity of 98%) from TCI Europe (Zwijndrecht, Belgium), potassium chloride (purity of 99.5%) from Fluka (Geel, Belgium), and potassium hydroxide (purity of 85%) from Merck (Darmstadt, Germany) were used without further purification. Double-distilled water was obtained using a Milli-Q Plus apparatus (Millipore, Milford, MA, USA). Deuterated water (99.9 at% D) was purchased from AstraChem (Saint-Petersburg, Russia).

Carboxymethylated CNCs from Cellulose Lab (Fredericton, NB, Canada) were used as received. According to the manufacturer, they have a density of 1.5 g/cm^3^, a degree of crystallinity close to 90% and a concentration of carboxylate groups of 1.2 mmol per 1 g of nanocrystals. The length and diameter of the CNCs under study are equal to 90 nm and 6 nm, respectively, as was previously determined by TEM and cryo-TEM [40].

### 4.2. Sample Preparation

At first, PO solutions were prepared. To do this, PO was dissolved in distilled water, and the pH was adjusted to 10.5 with 1M KOH. After that, KCl was added, and the mixture was stirred for 12 h. Once the PO solution had reached homogeneity, CNCs were added. The prepared mixtures were then stirred for several additional hours and sonicated for 5 min. In this study, the surfactant concentration was fixed at 3 wt%, while the CNC concentration was varied within a range of 0 to 8 wt%. Two different KCl concentrations were used: 1.3 and 2.6 wt%. At these concentrations, short and long PO micelles were formed, respectively.

Suspensions of pure CNCs were prepared by adding CNCs to aqueous KCl solution. The mixtures were stirred for 12 h and sonicated for 5 min. Similar to PO-CNC suspensions, the CNC suspensions contained 0–8 wt% CNCs at two salt concentrations: 1.3 and 2.6 wt% KCl.

Before analysis, all the samples were allowed to equilibrate for an additional 12 h. All the PO-CNC and CNC suspensions thus prepared were homogeneous and did not undergo phase separation for at least one month. Colloidal stability is due to the electrostatic repulsion between the PO micelles and the CNCs, as both the micelles [44] and nanoparticles [35] were fully ionized and likely charged under experimental conditions (pH = 10.5).

### 4.3. Rheology

The rheological characteristics were measured with the Anton Paar Physica MCR 301 rheometer (Anton Paar, Graz, Austria). A Peltier temperature controller was used to maintain the desired temperature during the experiment. All the samples were measured using a CP40-2 cone-plane cell (40 mm diameter and 2° cone angle). After loading the samples into the cell, they were left for 5 min. to reach equilibrium. The shear stress amplitude for the dynamic test was chosen to be within the range of linear viscoelastic response, where G′ and G″ are independent of the stress. All the experiments were performed in triplicate. In the continuous temperature increase test, each point was measured at a fixed frequency of 20 rad/s for 1 min, after a preliminary equilibration period of 3 min. at a given temperature. For the high-temperature experiments, a protective sample cover was used to minimize evaporation during the measurements.

### 4.4. Cryogenic Electron Microscopy

For structural analysis by cryogenic electron microscopy, samples were prepared on Lacey EM grids (Ted Pella, Northport, NY, USA). The grids were glow-discharged for 30 s at 0.26 mbar using a current of 20 mA with a PELCO easiGLOW (Ted Pella, Northport, NY, USA). For vitrification, 0.5 µL of the sample solution was applied to the grids. The grids were then blotted for 2.5 s and allowed to relax for 5 s using a Vitrobot Mark IV (Thermo Fisher Scientific, Waltham, MA, USA) at 20 °C and 100% humidity. Immediately after the relaxation step, the grids were plunged into liquid ethane. The grids were then transferred to a Gatan 626 side-entry cryo-holder (Gatan, Pleasanton, CA, USA) and maintained at liquid nitrogen temperature (−196 °C) during imaging. Images were recorded using a Tecnai Spirit microscope equipped with a LaB_6_ cathode (FEI Company, Hillsboro, OR, USA) operating at 120 kV, with a 1376 × 1024 pixel CCD Megaview camera. The estimation of the mesh size from the cryo-TEM micrographs was performed as described elsewhere [73].

### 4.5. Isothermal Titration Calorimetry

Nano ITC isothermal calorimeter (TA Instruments, New Castle, DE, USA) was applied to obtain thermodynamic parameters of titration of 0.05 wt% solution of PO into 0.3 wt% suspension of CNCs in the presence of 1.3 and 2.6 wt% of KCl at 20 °C. The raw thermograms were obtained by measuring the energy released while sequential injection of 2 μL of titrant into the measuring cell with 170 μL of substrate was performed with a 300 s time lag at a mixing rate of 200 rpm. The corresponding data of PO titration into the distilled water with the same salinity were subtracted from the raw thermograms to exclude the effect of micellar breakdown upon dilution (Figure S2). The thermodynamic profiles of enthalpy change with increasing molar ratio of PO molecules to CNCs were obtained by integration of the corrected thermograms (except for the first peak). To calculate the thermodynamic parameters of interaction between PO molecules and CNCs, the profiles were fitted with an independent binding site interaction model [74] in the NanoAnalyze 3.12.0 software (TA Instruments, New Castle, DE, USA).

### 4.6. Small-Angle Neutron Scattering

SANS studies were performed at the YuMO facility of the IBR-2 pulsed reactor in the framework of the user program of the Frank Laboratory of Neutron Physics at the Joint Institute for Nuclear Research (Dubna, Russia) in the range of neutron wavelengths from 0.05 to 0.8 nm. In the experiments, a time-of-flight diffractometer with a two-detector system [75], with the corresponding sample–detector distances of 4.5 and 12.96 m, was used. The dependences of the differential cross-section per sample volume on the scattering vector *Q* were obtained in the *Q* range of 0.007 to 0.4 Å^−1^. The samples were measured in disassemblable cylindrical cells with quartz walls, where the sample thickness was 2 mm. SANS measurements were conducted in D_2_O or in D_2_O/H_2_O mixture. Data from the ring-shaped detectors were processed using the SAS software (version 3.5.1) [76]. The normalization to a vanadium standard and the background corrections were applied.

### 4.7. Optical Microscopy

Optical microscopy studies were conducted using a Nikon Eclipse LV100 POL light microscope (Nikon, Tokyo, Japan) with a CFI TU Plan Fluor Epi objective lens (p 5×), at 20 and 60 °C. The samples were placed between two glass plates for measurements.

## Figures and Tables

**Table 1:** Enthalpy Δ*H*, entropy Δ*S* and Gibbs free energy Δ*G* changes, number of surfactant molecules per nanocrystal *n* and binding constants *K_a_* obtained from thermodynamic profiles of CNC’s titration with PO molecules at pH 10.5 and 20 °C upon fitting with independent binding sites model.

Concentration of KCl, wt%	Δ*H*, kJ/mol	*n*	*K_a_*,· 10^5^ mol^−1^	Δ*S*, J/mol·K	Δ*G*, kJ/mol
1.3	−5.4 ± 1.3	150 ± 19	3.1 ± 0.7	99	−34
2.6	−19.0 ± 1.3	290 ± 12	3.2 ± 1.8	41	−31

## Data Availability

Data is contained within the article or Supplementary Material.

## Supplementary Materials

The following supporting information can be downloaded at https://www.mdpi.com/article/10.3390/gels12060459/s1. Figure S1: Optical microscopy images of PO-CNC suspensions with compositions indicated in the figure at pH 10.5 and at different temperatures as noted in the figure; Figure S2: The thermograms of isothermal titration obtained by the gradual injection of 2 µL of 1.3 wt% aqueous solution of KCl with (a) and without 0.05 wt% PO (b) into 170 µL of 1.3 wt% aqueous solution of KCl at pH 10.5. Temperature: 20 °C; Figure S3: The thermograms of isothermal titration obtained by the gradual injection of 2 µL of 2.6 wt% aqueous solution of KCl with (a) and without 0.05 wt% PO (b) into 170 µL of 2.6 wt% aqueous solution of KCl at pH 10.5. Temperature: 20 °C; Figure S4: Cryo-TEM images of suspensions containing 3 wt% PO and 2 wt% CNCs. Solvent: 1.3 wt % KCl in water, pH 10.5; Figure S5: The ratio of the storage modulus $G^{\prime }$ of suspension containing 3 wt% PO and 2 wt% (hexagons) or 4 wt% CNCs (diamonds) to the storage modulus $G^{\prime }$ of 3 wt% solution of PO (both at a frequency of 20 rad/s) as a function of temperature. Solvent: 2.6 wt.% KCl in water, pH 10.5; Figure S6: Frequency dependences of storage (filled symbols) and loss (open symbols) moduli for suspensions containing 3 wt% PO and 2 wt% CNCs (a) or 4 wt% CNCs (b) at different temperatures indicated in the figure. Solvent: 2.6 wt% KCl in water, pH 10.5; Figure S7: Frequency dependences of storage (filled symbols) and loss (open symbols) moduli for (a) suspension containing 3 wt% PO and 2 wt% CNCs (circles) and (b) its components: 3 wt% solution of PO (squares) and 2 wt% suspension of CNCs (triangles) at 25 (blue), 40 (green) and 60 °C (red). Solvent: 2.6 wt% KCl in water, pH 10.5; Figure S8: SANS profile for 0.5 wt% suspension of CNCs. Solvent: 2.6 wt% KCl in D_2_O. Temperature: 25 °C; Figure S9: The raw thermogram (a) and corresponding binding isotherm (b) obtained by the gradual injection of 2 µL of aqueous solution containing 0.1 wt% PO and 2.6 wt% KCl into 170 µl of suspension containing 0.3 wt% CNCs and 2.6 wt% KCl at pH 10.5. Temperature: 60 °C; Figure S10: The thermogram of isothermal titration obtained by the gradual injection of 2 µL of aqueous solution containing 0.05 wt% PO and 2.6 wt% KCl into 170 µL of 2.6 wt% aqueous solution of KCl at pH 10.5. Temperature: 60 °C.
