# Supplementary material for: Temperature Tolerance of Self-Assembled Gels and Viscoelastic Solutions of Wormlike Micelles of Potassium Oleate Induced by Embedded Cellulose Nanocrystals
Source: Gels. 2026 May 24;12(6):459. doi: 10.3390/gels12060459 (PMC13297918; doi:10.3390/gels12060459)
Supplement: Supplementary file 1 [file gels-12-00459-s001.zip › gels-4289704-supplementary.pdf]

Supplementary Materials

for

Temperature Tolerance of Self-Assembled Gels and Viscoelastic Solutions of Wormlike Micelles of Potassium Oleate Induced by Embedded Cellulose Nanocrystals

Mikhail M. Avdeev, Vyacheslav S. Molchanov, Alexander L. Kwiatkowski, Yuri M. Chesnokov, Akhmed Kh. Islamov, Kuanysh Nazarov, and Olga E. Philippova*

3 wt% PO, 2 wt% CNC, 1.3 wt% KCl, 20 ^{0}C

3 wt% PO, 4 wt% CNC, 2.6 wt% KCl, 20 ^{0}C

3 wt% PO, 4 wt% CNC, 2.6 wt% KCl, 60 ^{0}C

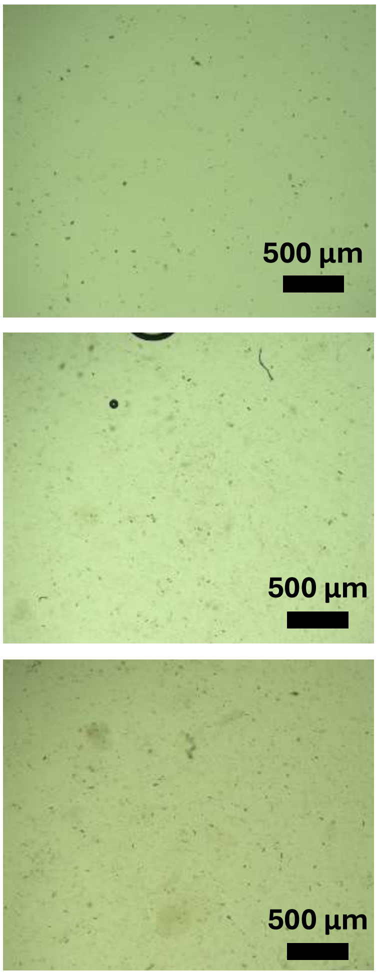

Figure S1. Optical microscopy images of PO-CNC suspensions with compositions indicated in the Figure at pH 10.5 and at different temperatures as noted in the Figure.

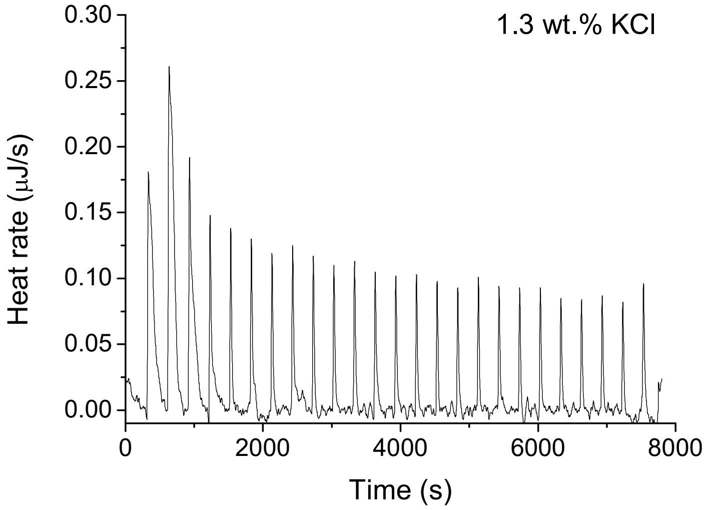

(**a**)

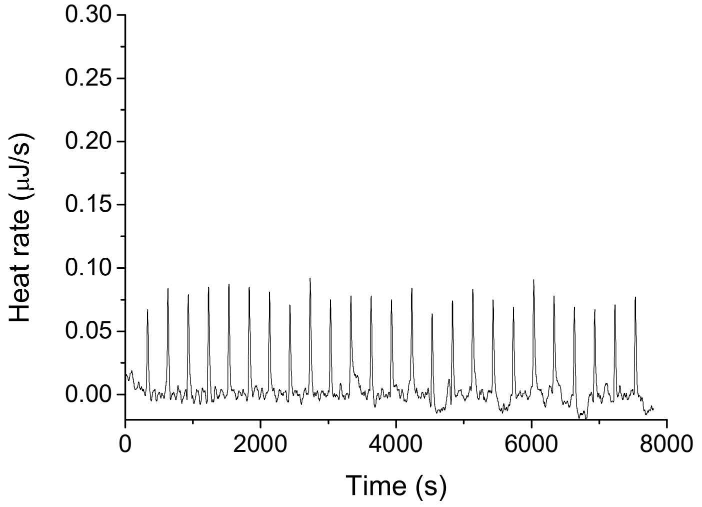

(**b**)

Figure S2. The thermograms of isothermal titration obtained by the gradual injection of 2 μL of 1.3 wt% aqueous solution of KCl with (**a**) and without 0.05 wt% PO (**b**) into 170 μL of 1.3 wt% aqueous solution of KCl at pH 10.5. Temperature: 20°C.

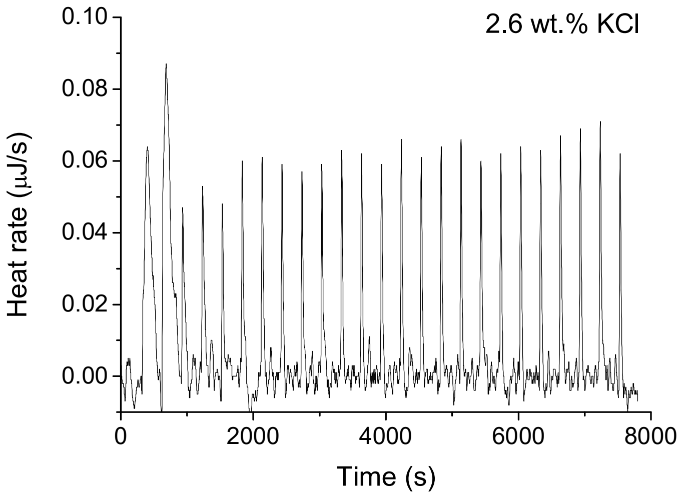

(**a**)

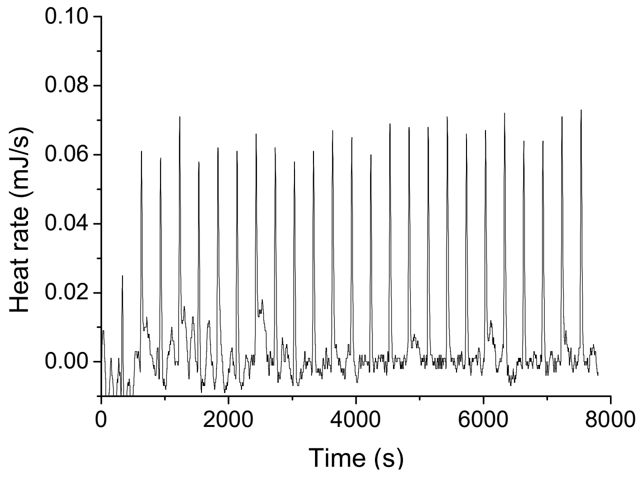

(**b**)

Figure S3. The thermograms of isothermal titration obtained by the gradual injection of 2 μL of 2.6 wt% aqueous solution of KCl with (a) and without 0.05 wt% PO (b) into 170 μL of 2.6 wt% aqueous solution of KCl at pH 10.5. Temperature: 20°C.

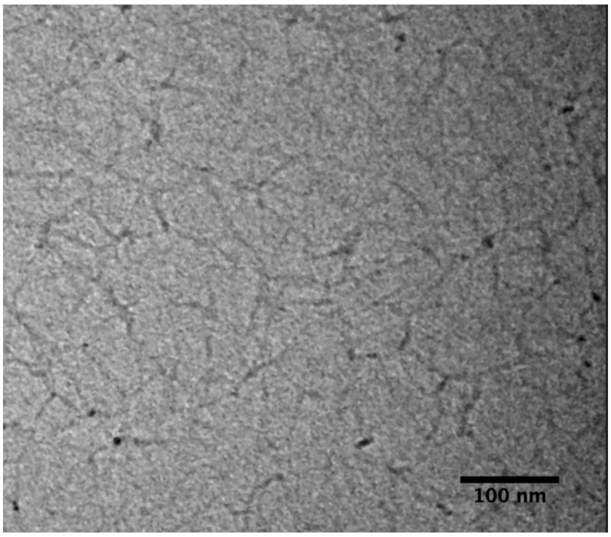

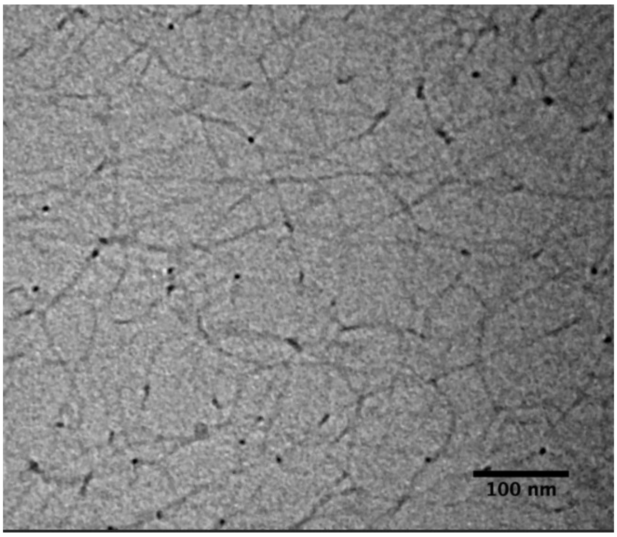

(**a**) (**b**)

Figure S4. Cryo-TEM images of suspensions containing 3 wt% PO and 2 wt% CNCs. Solvent: 1.3 wt % KCl in water, pH 10.5.

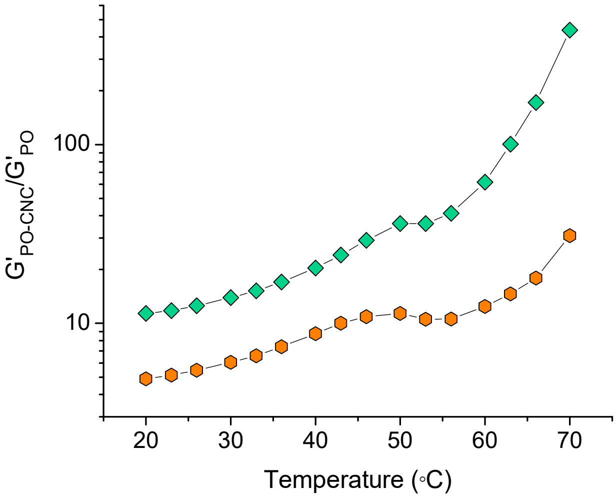

Figure S5. The ratio of the storage modulus G' of suspension containing 3 wt% PO and 2 wt% (hexagons) or 4 wt% CNCs (diamonds) to the storage modulus G' of 3 wt% solution of PO (both at a frequency of 20 rad/s) as a function of temperature. Solvent: 2.6 wt.% KCl in water, pH 10.5.

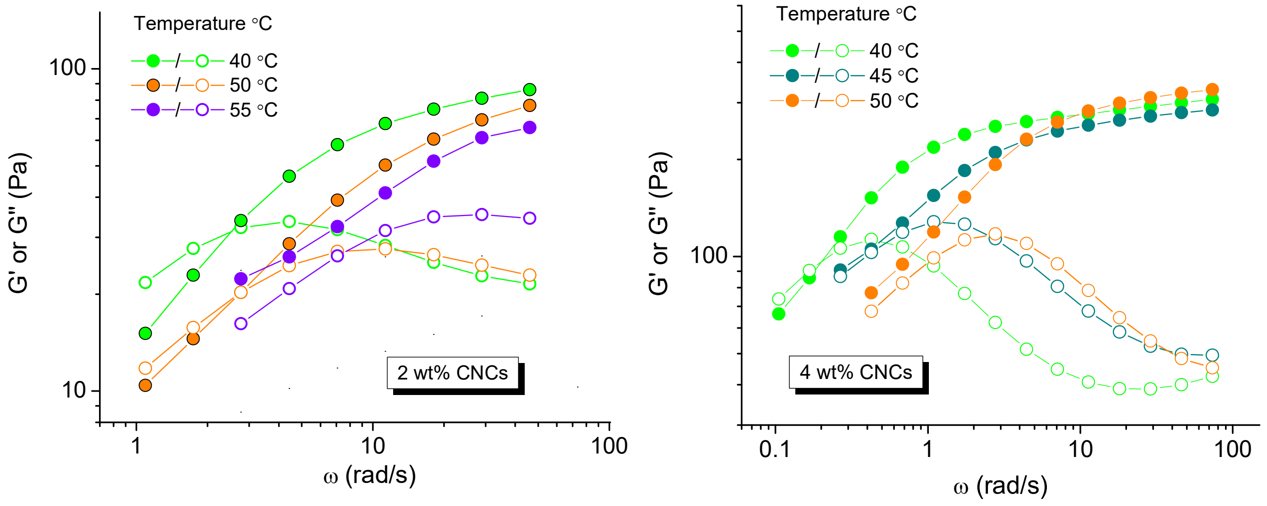

(**a**) (**b**)

Figure S6. Frequency dependences of storage (filled symbols) and loss (open symbols) moduli for suspensions containing 3 wt% PO and 2 wt% CNCs (a) or 4 wt% CNCs (b) at different temperatures indicated in the Figure. Solvent: 2.6 wt% KCl in water, pH 10.5.

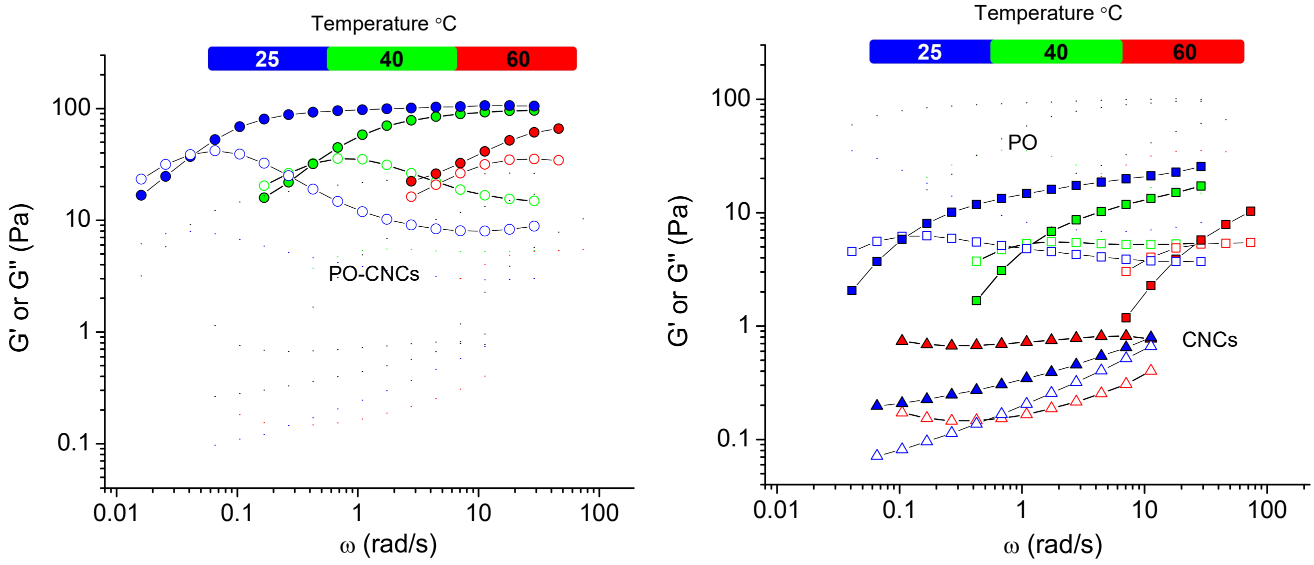

(**a**) (**b**)

Figure S7. Frequency dependences of storage (filled symbols) and loss (open symbols) moduli for (**a**) suspension containing 3 wt% PO and 2 wt% CNCs (circles) and (**b**) its components: 3 wt% solution of PO (squares) and 2 wt% suspension of CNCs (triangles) at 25 (blue), 40 (green) and 60 °C (red). Solvent: 2.6 wt% KCl in water, pH 10.5.

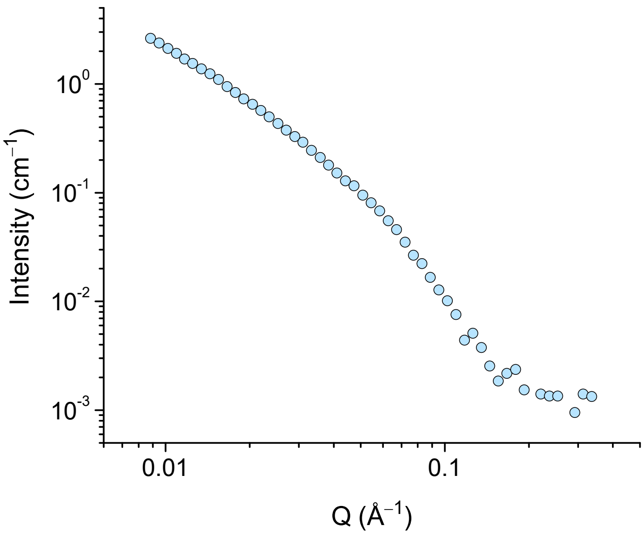

Figure S8. SANS profile for 0.5 wt% suspension of CNCs. Solvent: 2.6 wt% KCl in D_2O. Temperature: 25°C.

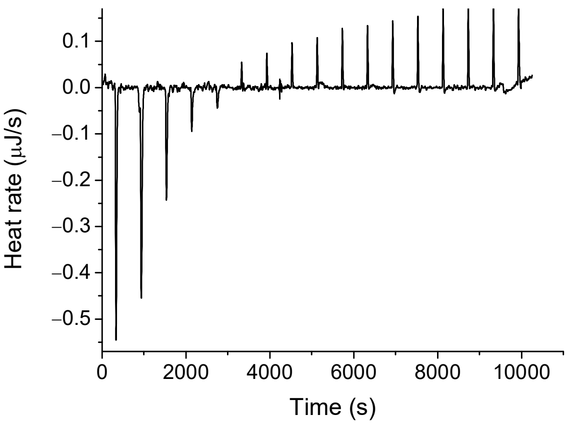

(**a**)

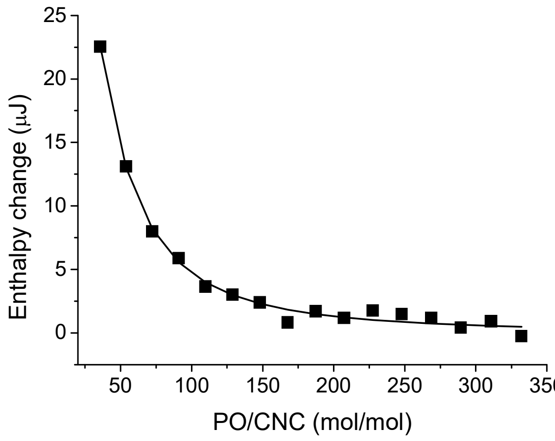

(**b**)

Figure S9. The raw thermogram (**a**) and corresponding binding isotherm (**b**) obtained by the gradual injection of 2 μL of aqueous solution containing 0.1 wt% PO and 2.6 wt% KCl into 170 μL of suspension containing 0.3 wt% CNCs and 2.6 wt% KCl at pH 10.5. Temperature: 60°C.

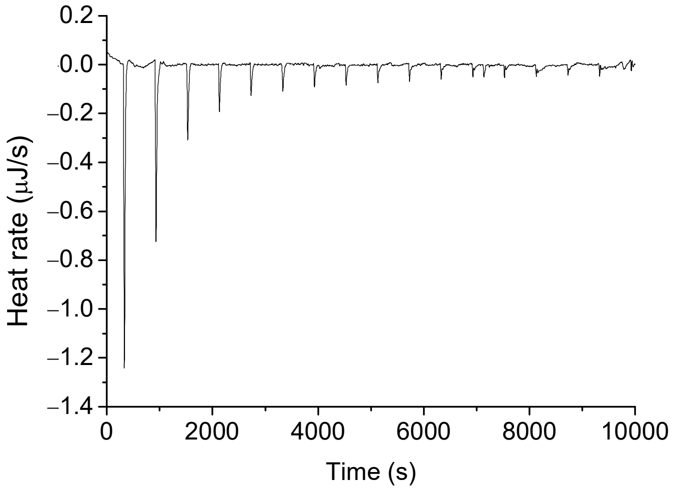

Figure S10. The thermogram of isothermal titration obtained by the gradual injection of 2 μL of aqueous solution containing 0.05 wt% PO and 2.6 wt% KCl into 170 μL of 2.6 wt% aqueous solution of KCl at pH 10.5. Temperature: 60°C.
